# Thioredoxin-Interacting Protein (TXNIP) Knockdown Protects against Sepsis-Induced Brain Injury and Cognitive Decline in Mice by Suppressing Oxidative Stress and Neuroinflammation

**DOI:** 10.1155/2022/8645714

**Published:** 2022-05-05

**Authors:** Yu Zhang, Cheng-Jun Xing, Xiao Liu, Ya-Hong Li, Jing Jia, Jian-Guo Feng, Cheng-Jie Yang, Ye Chen, Jun Zhou

**Affiliations:** ^1^Department of Anesthesiology, The Affiliated Hospital of Southwest Medical University, Luzhou, China; ^2^Laboratory of Anesthesiology, Southwest Medical University, Luzhou, China; ^3^Department of Traditional Chinese Medicine, The Affiliated Hospital of Southwest Medical University, Luzhou, China

## Abstract

Sepsis-associated encephalopathy (SAE) is linked to increased morbidity and mortality rates in patients with sepsis. Increased cytokine production and neuronal apoptosis are implicated in the pathogenesis of the SAE. Neuroinflammation plays a major role in sepsis-induced brain injury. Thioredoxin-interacting protein (TXNIP), an inhibitor of thioredoxin, is associated with oxidative stress and inflammation. However, whether the TXNIP is involved in the sepsis-induced brain injury and the underlying mechanism is yet to be elucidated. Therefore, the present study was aimed at elucidating the effects of TXNIP knockdown on sepsis-induced brain injury and cognitive decline in mice. Lipopolysaccharide (LPS) was injected intraperitoneally to induce sepsis brain injury in mice. The virus-carrying control or TXNIP shRNA was injected into the lateral ventricle of the brain 4 weeks before the LPS treatment. The histological changes in the hippocampal tissues, encephaledema, and cognitive function were detected, respectively. Also, the 7-day survival rate was recorded. Furthermore, the alterations in microglial activity, oxidative response, proinflammatory factors, apoptosis, protein levels (TXNIP and NLRP3 inflammasome), and apoptosis were examined in the hippocampal tissues. The results demonstrated that the TXNIP and NLRP3 inflammasome expression levels were increased at 6, 12, and 24 h post-LPS injection. TXNIP knockdown dramatically ameliorated the 7-day survival rate, cognitive decline, brain damage, neuronal apoptosis, and the brain water content, inhibited the activation of microglia, downregulated the NLRP3/caspase-1 signaling pathway, and reduced the oxidative stress and the neuroinflammatory cytokine levels at 24 h post-LPS injection. These results suggested a crucial effect of TXNIP knockdown on the mechanism of brain injury and cognitive decline in sepsis mice via suppressing oxidative stress and neuroinflammation. Thus, TXNIP might be a potential therapeutic target for SAE patients.

## 1. Introduction

Sepsis is a life-threatening complication of severe infection, leading to tissue damage, multiple organ failure, and death if it is not treated promptly [1–3]. Among the multiple organs affected, brain damage occurs early in severe sepsis and promotes the development of other signs and symptoms. Brain damage is manifested by a series of neurological dysfunctions, such as delirium, coma, seizures, and focal neurological symptoms, causing sepsis-associated encephalopathy (SAE) [4–9]. According to previous reports, SAE occurs in about 70% of patients with sepsis, causing poor outcomes in the intensive care unit (ICU) [7, 9, 10]. The SAE is related to increased mortality of patients with sepsis [11, 12]. In addition, the SAE may cause permanent cognitive dysfunction, increase the susceptibility of the brain to neurodegenerative diseases, and increase the risk of developing dementia [13]. However, the mechanism of brain damage caused by sepsis is yet inconclusive. Currently, there is a lack of specific drugs for SAE treatment. Treating infection, organ failure, and metabolic disorders while avoiding the use of neurotoxic drugs is adopted for managing sepsis [8, 14, 15]. Thus, exploration of the mechanism of SAE will develop specific new therapeutic strategies.

Neuroinflammation and oxidative stress are manifested in various neurological diseases [16–18]. As one of the pattern recognition receptors, the nucleotide-binding oligomerization domain-like receptor protein 3 (NLRP3) inflammasome is a supramolecular complex composed of NLRP3, apoptosis-associated speck-like protein (ASC), and caspase-1, which are indispensable for the body's innate immunity against microbial infection [19]. The complex is enriched in immune cells, including macrophages and microglia, and participates in the progress of inflammatory response. The NLRP3 inflammasome functions via caspase-1 zymogen activation, causing the proteolytic activation of proinflammatory cytokines, IL-1*β* and IL-18. A previous study using a mouse model showed that the NLRP3/caspase-1 pathway is involved in SAE. Moreover, the application of the NLRP3 inhibitor MCC950 effectuated NLRP3-mediated neuronal pyrolysis and proinflammatory cytokines [20]. However, the mechanism underlying the NLRP3 activation has not yet been elucidated.

The thioredoxin (TRX) system is widely present in the body and functions as a critical antioxidant. TRX is the main member of the TRX system and eliminates reactive oxygen species (ROS) by reducing the thiols [21, 22]. Thioredoxin-interacting protein (TXNIP) is a 46 kDa protein that binds to TRX, inhibits the antioxidant effect of TRX, and controls the cellular redox state [23, 24]. TXNIP plays a critical role in cerebrovascular and neurodegenerative diseases, such as subarachnoid hemorrhage and Alzheimer's disease [23, 25]. However, no study has been reported on whether TXNIP is involved in septic brain injuries.

In recent years, the cause-and-effect correlation between oxidative stress and the inflammatory response has gained increasing attention. Oxidative stress and inflammatory response are closely related to the pathogenesis of SAE. Oxidative stress can aggravate proinflammatory cytokine expression. Recent studies have shown that the TXNIP binds to NLRP3 and activates the NLRP3 inflammasome under excessive oxidative stress [24, 26]. Subsequently, the effect of TXNIP on the NLRP3 inflammasome has been observed in animal models, such as renal ischemia-reperfusion injury and myocardial ischemia-reperfusion injury models [27, 28]. Thus, it could be deemed that TXNIP is the modulator between oxidative stress and inflammation. However, specific research on the role of the TXNIP/NLRP3 signaling pathway in septic brain injury is yet lacking.

Consequently, the present study was aimed at evaluating the effects of TXNIP knockdown on sepsis-induced brain injury and cognitive impairment and the related molecular mechanism. Herein, we proposed that TXNIP knockdown ameliorates brain injury and cognitive decline after sepsis by regulating oxidative stress and neuroinflammation.

## 2. Materials and Methods

### 2.1. Animals

The current study protocol was approved by the Animal Care Committee of the Southwest Medical University, Luzhou, China (approval number: 20180306042). All procedures were performed following the National Institutes of Health guidelines for the use of experimental animals. A total of 518 male C57BL*/*6 mice (6–8 weeks), weighing 18–22 g, were purchased from Chengdu Dossy Experimental Animals Co., Ltd., China. The animals were housed in cages in a temperature-controlled room (22 ± 2°C) with an alternating 12 h light/dark cycle and acclimated for a week before the study.

### 2.2. Experimental Design

**Figure 1:**
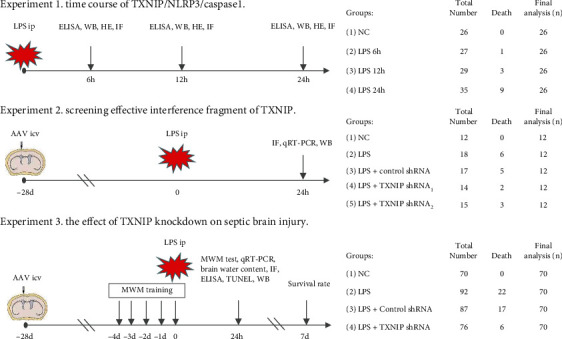
Experimental design and animal groups. AAV: adeno-associated virus; LPS: lipopolysaccharide; ip: intraperitoneal injection; icv: lateral cerebral ventricle; TXNIP: thioredoxin-interacting protein; NLRP3: nucleotide-binding oligomerization domain-like receptor protein 3; MWM: Morris water maze; HE: hematoxylin-eosin staining; WB: Western blotting; IF: immunofluorescence; qRT-PCR: quantitative real-time polymerase chain reaction; TUNEL: terminal deoxynucleotidyl transferase dUTP nick end labeling; ELISA: enzyme-linked immunosorbent assay.

In the current study, all mice were randomly assigned to the following three experiments (Figure 1).

#### 2.2.1. Experiment 1

To evaluate the injury and endogenous TXNIP, NLRP3, and cleaved caspase-1 temporal levels in the hippocampus of septic mice, 104 surviving animals were randomly divided into four groups (*n* = 26/group): negative control (NC) and 6, 12, and 24 h after lipopolysaccharide (LPS) treatment. The mice in the sepsis groups were injected intraperitoneally with 15 mg/kg LPS (Sigma-Aldrich, St. Louis, MO, USA), while the NC group was injected with an equivalent volume of normal saline. Western blotting, hematoxylin-eosin (HE) staining, enzyme-linked immunosorbent assay (ELISA), and Iba1 immunofluorescence staining were conducted at the corresponding time points after the LPS injection.

#### 2.2.2. Experiment 2

To screen the most effective interference fragment of TXNIP, 60 surviving mice were randomly divided into five groups (*n* = 12/group): NC, LPS, LPS+control shRNA, LPS+TXNIP shRNA_1_, and LPS+TXNIP shRNA_2_. A 5 *μ*L virus-carrying control shRNA, TXNIP shRNA_1_, or TXNIP shRNA_2_ was injected into the lateral cerebral ventricle in the LPS+control shRNA, LPS+TXNIP shRNA_1_, and LPS+TXNIP shRNA_2_ groups, respectively. The shRNA sequences are listed in the following: TXNIP shRNA_1_ (5′-GCAAACAGACTTTGGACTACTCGAGTAGTCC-AAAGTCTGTTTGC-3′) and TXNIP shRNA_2_ (5′-GCTGGATAGACCTAAACATCT- CGAGATGTTTAGGTCTATCCAGC-3′). The control shRNA sequences are as follows: 5′-CCTAAGGTTAAGTCGCCCTCGCTCGAGCGAGGGCGACTTAACC-TTAGG-3′. After 4 weeks, a sepsis model was established by intraperitoneal injection of 15 mg/kg LPS or the same volume of normal saline. Western blotting and quantitative real-time polymerase chain reaction (qRT-PCR) were performed 24 h post-LPS injection.

#### 2.2.3. Experiment 3

To observe the effect of TXNIP knockdown on the injury and NLRP3/caspase-1 pathway in the hippocampus of septic mice, 280 enrolled mice were randomly divided into four groups (*n* = 70/group): NC, LPS, LPS+control shRNA, and LPS+TXNIP shRNA. A volume of 5 *μ*L adeno-associated virus (AAV) vectors containing control shRNA or TXNIP shRNA_1_ was injected into the lateral ventricle of the brain in the LPS+control shRNA or LPS+TXNIP shRNA group, respectively. After 4 weeks, a sepsis model was established by the intraperitoneal injection of 15 mg/kg LPS or the equivalent volume of normal saline. For the survival study, 20 mice from each group were selected randomly to observe the survival rate for 7 days. In addition, on the 4th day (-4 d) before modeling, 10 mice from each group were selected randomly for the water maze experiment. Subsequently, another 40 mice were sacrificed 24 h after modeling for the brain water content, Western blotting, qRT-PCR, enzyme-linked immunosorbent assay (ELISA), hematoxylin-eosin (HE) staining, Iba1 immunofluorescence staining, and terminal deoxynucleotidyl transferase dUTP nick end-labeling (TUNEL) staining.

### 2.3. Sepsis Model

Animals in the LPS group received an intraperitoneal injection (ip) of LPS (15 mg/kg) isolated from *Escherichia coli* O111:B4 and diluted in sterile normal saline, while the NC group received an equal volume of normal saline. Since LPS induced a high mortality rate, mice that died during the experiment (except for the survival study) were excluded and relevant mice were supplemented as previously described [29–31].

### 2.4. Specimen Preparation

At the corresponding time after the LPS injection, mice were sacrificed under anesthesia and the blood was collected from eyes for the ELISA test. The whole brains were removed immediately and postfixed for 24 h. The sections were then prepared for HE staining, immunohistochemical staining, and the TUNEL assay. Moreover, the hippocampal CA1 region was rapidly microdissected from both sides of the hippocampal fissure by sharp dissection on ice, frozen in liquid nitrogen, and stored at -80°C for subsequent testing as previously described [32, 33].

### 2.5. AAV Injection

The AAV carried the enhanced green fluorescent protein (*EGFP*) gene. The AAV injection was administered on day 28 before the LPS or normal saline injection, as described previously [34]. Briefly, the AAV vectors (AAV-control shRNA, AAV-TXNIP shRNA_1_, and AAV-TXNIP shRNA_2_) were constructed by Yunzhou Biosciences Co., Ltd. (Guangzhou, China). Before exposing the skull, the mice were anesthetized with pentobarbital sodium using a stereotaxic apparatus (RWD, Shenzhen, China). A volume of 5 *μ*L AAV vectors was injected stereotaxically into the bilateral ventricles (AP -0.3 mm; ML ±1.0 mm; DV 3.0 mm) at a rate of 0.5 *μ*L/min. The needle was kept in place for 5 min and screwed out slowly. The skin incision was sutured, and the mice were housed in a single cage after the recovery from anesthesia.

### 2.6. 7-Day Survival Rate

The survival of mice in each group was recorded daily after modeling, and the observation was continued for 7 days.

### 2.7. Morris Water Maze (MWM) Test

The function of spatial learning and memory for mice was assessed using the MWM test. The maze (Shanghai XinRuan Information Technology Co. Ltd., Shanghai, China) consisted of a round tank (height 50 cm and diameter 120 cm), a platform (diameter 10 cm), and a camera analysis system [35]. The mice were trained for four trials per day for 5 consecutive days (-4 d to 0 d) before modeling. In every trial, the mice were placed on a different start quadrant and allowed to find a platform submerged 1 cm below the water surface within a maximum time of 60 s, and the escape latency was recorded. Animals that failed to locate the platform within 60 s were manually guided to the platform. On the fifth day (0 d) after MWM training, the mice were injected LPS or saline intraperitoneally. The water maze test was performed on the sixth day; namely, at 24 hours after modeling (24 h), the mice were placed into the water facing the pool wall to navigate freely in the water for 60 s, and the escape latency and the swimming speed were recorded. Then, the platform was withdrawn, and the mice were allowed to navigate freely in the water again for 60 s. During the trial, the percentage of retention time in the target quadrant and the times of crossing the former platform were recorded as we previously described [30, 35, 36].

### 2.8. Brain Water Content Measurement

The brain water content was estimated by the wet-to-dry brain weight ratio, as described previously [36]. Briefly, the whole brains were harvested under deep anesthesia and immediately weighed to obtain the wet weight. Then, the brain tissues were dried at 65°C for 48 h to obtain the dry weight. Brain water content = (wet weight − dry weight)/wet weight × 100%.

### 2.9. HE Staining

The whole brain tissues were fixed in 4% paraformaldehyde, embedded in paraffin, and cut into 3 *μ*m thick hippocampus coronal sections. The tissue sections were stained with HE and observed under a light microscope (Olympus, Tokyo, Japan) by two experienced pathologists blinded to the study. The number of normal neurons per square millimeter (cells/mm^2^) in 6 brain sections of each mouse was calculated according to the size of the CA1 subregion in four randomly selected high-magnification fields (×400) of each section and averaged as we previously described [36].

### 2.10. TUNEL Staining

Fixed brain tissue was dehydrated by sucrose gradient solutions, and frozen sections were collected after the optimal cutting temperature (OCT) medium embedding. The apoptosis of neural cells in the hippocampus was detected by the TUNEL method, following the manufacturer's instructions (Roche, Basel, Switzerland). The TUNEL reagents of terminal deoxynucleotidyl transferase (TdT) enzyme reaction solution and tetramethylrhodamine (TMR) labeling solution at a ratio of 1 : 9 were added to the sections for 1 h. The nuclei were stained with 4′,6-diamidino-2-phenylindole (DAPI) (Solarbio, Beijing, China) for 5 min. The stained tissue sections were observed under a fluorescence microscope (Olympus), and the images were captured. The TUNEL-positive cell percentage (%) was calculated as follows: TUNEL‐positive cells/total cells × 100% [36].

### 2.11. Immunofluorescence Staining

The protein expression of ionized calcium binding adaptor molecule 1 (Iba1) reflecting the activation of microglia was detected by the immunofluorescence method [36]. Briefly, fixed brain tissue was dehydrated by sucrose gradient solutions, and the frozen sections were stained after OCT medium embedding. Subsequently, the tissue sections were fixed in paraformaldehyde for 30 min, permeabilized with 0.2% Triton X-100 for 15 min, blocked in 1% bovine serum albumin for 30 min, and incubated with primary antibody rabbit anti-Iba1 (1 : 100, Cell Signaling Technology (CST), Danvers, MA, USA) at 4°C overnight. After washing, the sections were incubated with the corresponding FITC-labeled or Cy3-labeled secondary antibody (Kangwei Century Biotechnology Co., Ltd., Beijing, China) in the dark at 37°C for 60 min. Finally, the tissue sections were incubated for 5 min with DAPI before examining the protein levels under a fluorescence microscope. For quantification, the HE mean number of Iba1-positive cells in hippocampal CA1 area was calculated. Activated microglia were calculated as the percentage of cells with shorter, stubbier processes, namely, activated microglia% = (the number of activated microglia/total number of Iba1‐positive cells) × 100 [36].

### 2.12. qRT-PCR

Total RNA was extracted from the hippocampal tissues using an RNAsimple total RNA kit (Tiangen, Beijing, China), following the manufacturer's protocol. The isolated RNA was reverse transcribed into cDNA using the ReverTra Ace qPCR RT Master Mix (Toyobo Co., Ltd., Osaka, Japan), according to the manufacturer's protocol. Then, the cDNA was amplified using SuperReal PreMix Plus (SYBR Green) (Tiangen, Beijing, China) on a real-time PCR system (Roche). The amplification parameters were as follows: 95°C for 15 min, followed by 5 cycles of 95°C for 10 s and 60°C for 32 s. *β*-Actin was used as an internal reference for the quantification of the TXNIP gene expression level. The normalized mRNA expression level in the control group (target mRNA/*β*-actin value) was utilized to calculate the fold changes of the mRNA levels in the other groups. The primer sequences are as follows: TXNIP (forward: 5′-ATACTCCTTGCTGATCTACG-3′, reverse: 5′-TGGGGTATCTGGG-ATGTTTA-3′) and *β*-actin (forward: 5′-TTTGCAGCTCCTTCGTTGC-3′, reverse: 5′-TCGTCATCCATGGCGAACT-3′).

### 2.13. ROS, MDA, GSH-Px, and SOD Level Measurement

Supernatants from hippocampal tissues were obtained, and specific kits (Nanjing Jiancheng Biotechnology Research Institute, Nanjing, China) were used to measure the ROS and MDA levels and GSH-Px and SOD activities, according to the manufacturer's instructions and previous studies [37, 38].

### 2.14. ELISA

The hippocampal tissue homogenates were prepared, and supernatants were collected by centrifugation of the lysates at 12000 rpm, 4°C for 15 min. The TNF-*α*, IL-6, IL-1*β*, and IL-18 levels were estimated in the hippocampal tissues using the ELISA kits (Meimian, Wuhan, China), following the manufacturer's instructions and as described previously [37, 39].

### 2.15. Western Blotting Analysis

The hippocampal tissue was excised and placed on ice for 30 min before lysis, followed by ultracentrifugation at 4°C. The protein concentration of the supernatant was measured by using the BCA kit (Beyotime, Shanghai, China). An equivalent of 25 *μ*g protein was separated by sodium dodecyl sulfate-polyacrylamide gel electrophoresis (SDS-PAGE), transferred to a polyvinylidene fluoride membrane (Amersham Biosciences, NJ, USA), or a nitrocellulose membrane (Beyotime), blocked with 5% skimmed milk for 2 h, and probed with the following primary antibodies at 4°C overnight: anti-TXNIP (1 : 1000, CST), anti-NLRP3 (1 : 1000, CST), anti-caspase-1 (1 : 200, Santa Cruz Biotech, Santa Cruz, CA, USA), anti-Bcl-2 (1 : 1000, Abcam, Cambridge, MA, USA), anti-Bax (1 : 1000, Proteintech, China), anti-cleaved caspase-3 (1 : 500, Proteintech), and anti-*β*-actin (1 : 5000, Proteintech). Subsequently, the membrane was incubated with the horseradish peroxidase-labeled goat anti-rabbit or goat anti-mouse secondary antibody (1 : 1000, Solarbio) at 37°C for 1 h. Finally, the membrane was exposed to enhanced chemiluminescence, and the immunoreactive bands were quantitated using the ImageJ software (version 1.31; National Institutes of Health, Bethesda, MD, USA) [30].

### 2.16. Statistical Analysis

GraphPad Prism 8.3 statistical software (GraphPad Software, San Diego, CA, USA) was used for all statistical analyses. The data were expressed as the mean and standard error of the mean (mean ± SEM). One-way analysis of variance (ANOVA) and Tukey's post hoc test were used for comparisons. For the training phase of the MWM test, the escape latency over time was analyzed by two-way repeated measures ANOVA [36]. Kaplan-Meier survival curves were analyzed using the log-rank test. *P* < 0.05 indicated statistically significant difference.

## 3. Results

### 3.1. Effects of LPS on Brain Injury in Mice

**Figure 2:**
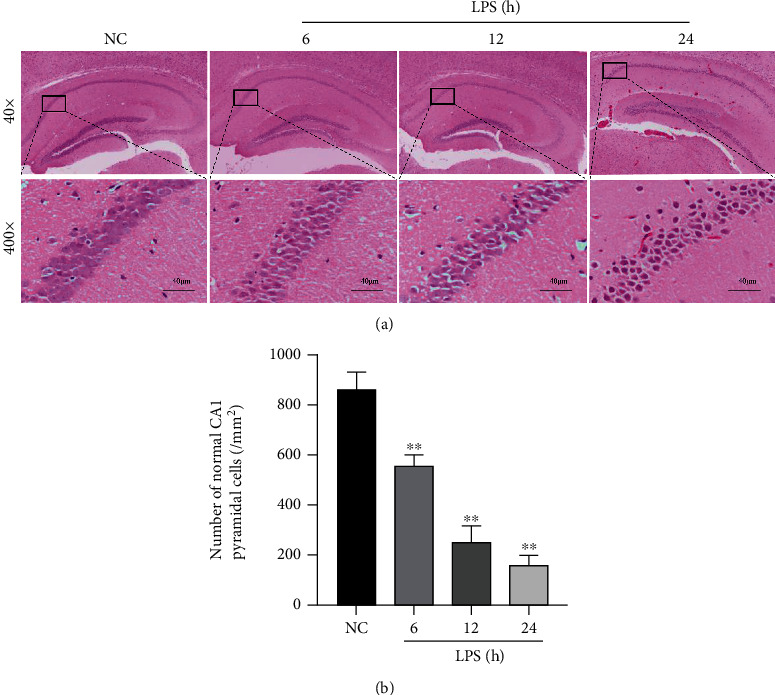
LPS caused histological injury to the hippocampus. (a) HE staining of the hippocampal CA1 region (×400) (scale bar = 40 *μ*m). (b) Statistics of the normal pyramidal cells in the hippocampal CA1 area. The data are presented as the mean ± SEM. ^∗∗^*P* < 0.01*vs*. NC group (*n* = 8).

The HE staining showed that the cone cells in the hippocampal CA1 area in the NC group were arranged in an orderly manner and evenly distributed with obvious nucleoli, while in the LPS group, the cells were arranged disorderly and were morphologically irregular, the cytoplasm was deeply stained, and the nuclei were constricted (Figure 2(a)). Compared to the NC group, the number of normal pyramidal cells in the hippocampal CA1 area in the LPS group was reduced and further minimized in the LPS 24 h group (*P* < 0.01) (Figure 2(b)).

### 3.2. Changes in the Inflammatory Cytokine Levels in the Serum and Hippocampus

**Figure 3:**
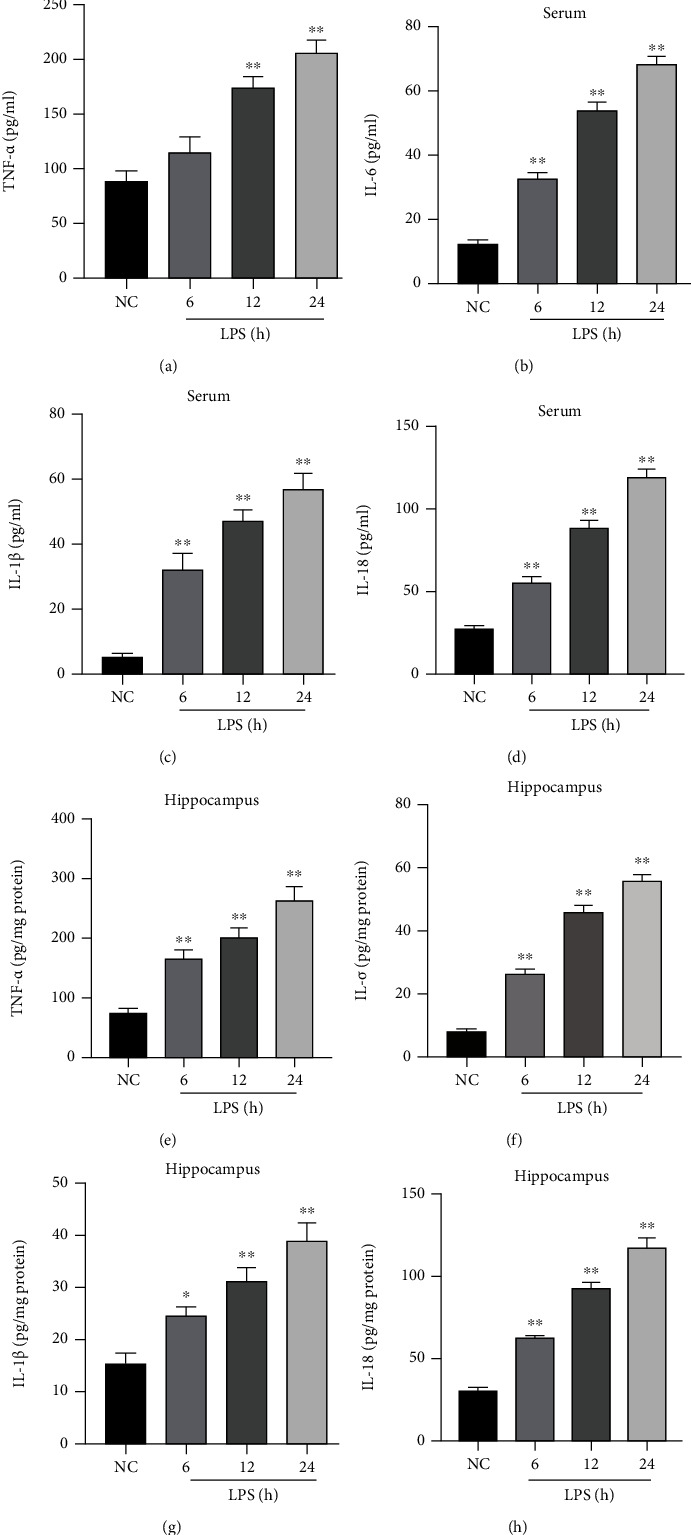
Changes in the inflammatory factors in the serum and hippocampus. (a–d) The levels of TNF-*α*, IL-6, IL-1*β*, and IL-18 in the serum, respectively. (e–h) The levels of TNF-*α*, IL-6, IL-1*β*, and IL-18 in the hippocampus, respectively. The values are presented as mean ± SEM. ^∗^*P* < 0.05, ^∗∗^*P* < 0.01*vs*. NC group (*n* = 6).

As presented in Figure 3, compared to the NC group, the levels of TNF-*α*, IL-6, IL-1*β*, and IL-18 in the serum (Figures 3(a)–3(d)) and hippocampus (Figures 3(e)–3(h)) were increased significantly in the LPS 6 h, LPS 12 h, and LPS 24 h groups and reached the peak in the LPS 24 h group (*P* < 0.05 or *P* < 0.01).

### 3.3. Effects of LPS on Microglial Activation in the Hippocampus of Mice

**Figure 4:**
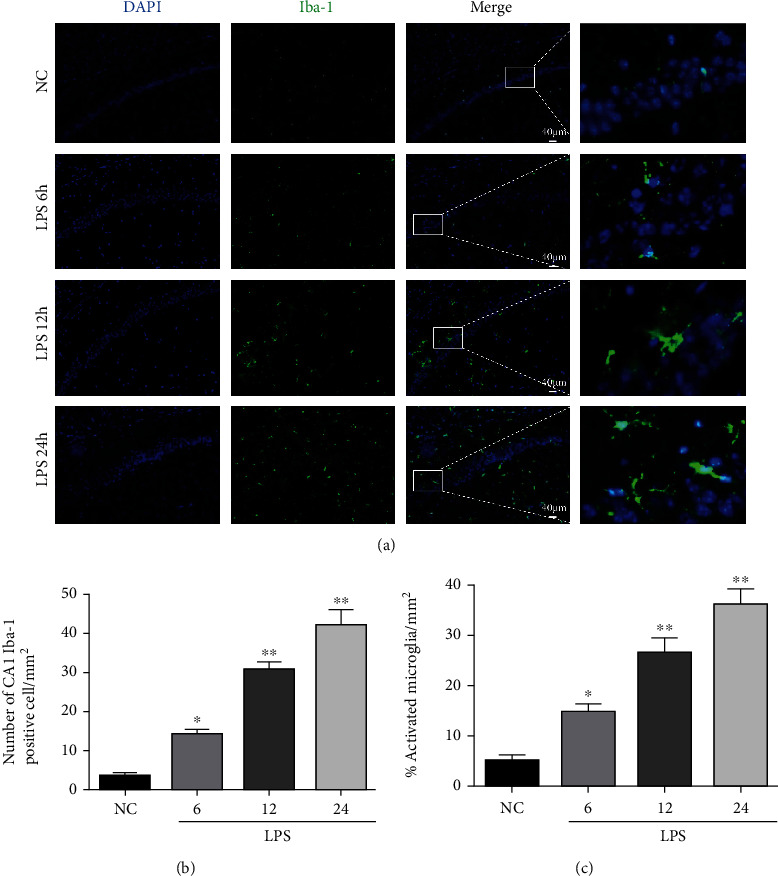
Changes in Iba1-positive cells in the hippocampal CA1 region after LPS injection. (a) Immunofluorescence of the microglia in the hippocampal CA1 region (×100) (scale bar = 40 *μ*m). Nuclei were labeled with blue fluorescence (DAPI), and Iba1 was labeled with green fluorescence. (b, c) Quantitative analysis of Iba1-positive cells and activated microglia in the hippocampal CA1 region. The data are presented as the mean ± SEM. ^∗^*P* < 0.05, ^∗∗^*P* < 0.01*vs*. NC group (*n* = 6).

The microglia cell was small, and the synapses were slender and scattered in the NC group. Compared to the NC group, a significantly increased number of Iba1-positive cells and the percentage of activated microglia indicated by enlarged cell bodies and shortened synapse were found in the hippocampal CA1 area after the LPS injection, especially in the LPS 24 h group (Figures 4(a)–4(c)).

### 3.4. Effects of LPS on the Expression of TXNIP and NLRP3 Inflammasome

**Figure 5:**
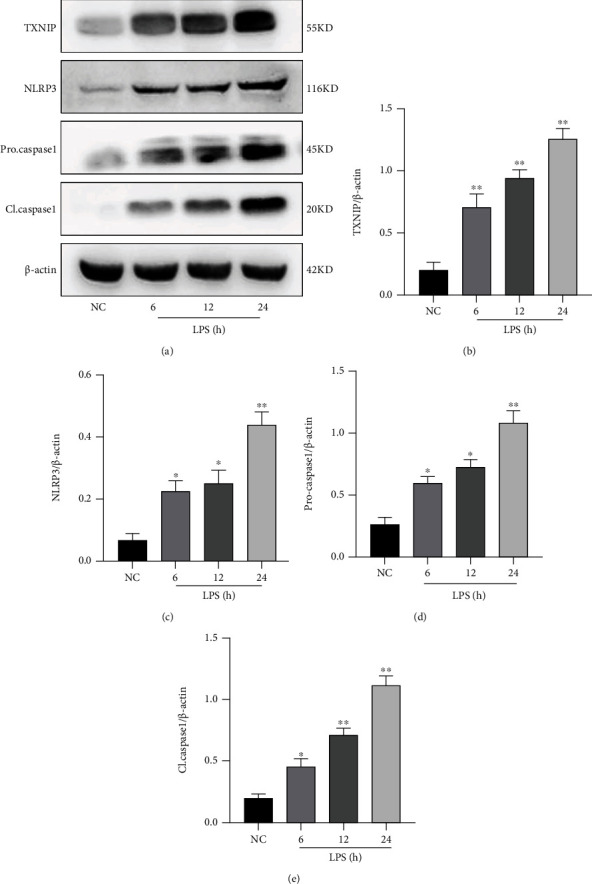
Expression of the TXNIP-NLRP3 signaling pathway in the hippocampus. (a) Bands of the TXNIP, NLRP3, procaspase-1, and cleaved caspase-1. (b–e) Quantification of the expression levels of TXNIP, NLRP3, procaspase-1, and cleaved caspase-1. The data are presented as mean ± SEM. ^∗^*P* < 0.05, ^∗∗^*P* < 0.01*vs*. NC group (*n* = 6).

The Western blot results showed (Figure 5) that the expression level of TXNIP, NLRP3, procaspase-1, and cleaved caspase-1 in the hippocampus in the LPS group was significantly increased compared to that in the NC group at 6, 12, and 24 h after LPS injection (*P* < 0.05 or *P* < 0.01). These results suggested that LPS could cause the activation of the TXNIP-NLRP3 signaling pathway.

### 3.5. AAV-TXNIP shRNA Inhibited the Expression of TXNIP in the Hippocampus

**Figure 6:**
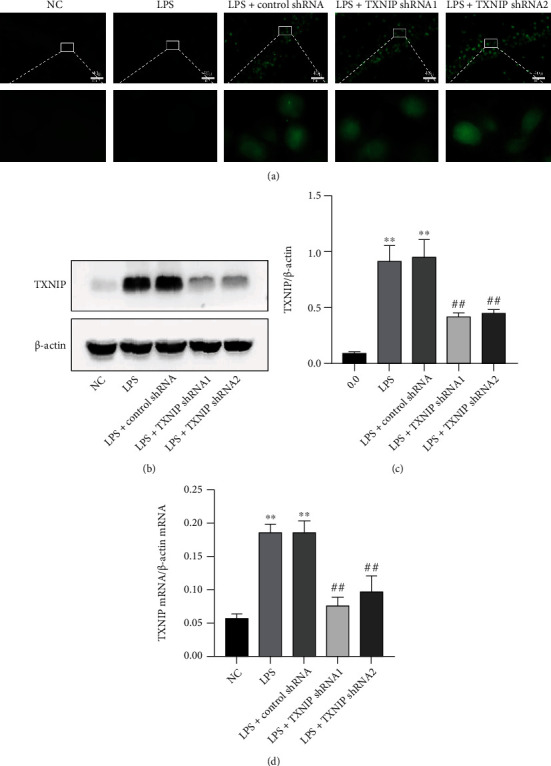
Effect of AAV-TXNIP shRNA on the expression of TXNIP. After transfection of TXNIP shRNA with the AAV carrier, the expression of TXNIP was detected in the hippocampus. (a) The AAV expresses EGFP (green) in the hippocampus (×400) (scale bar = 40 *μ*m). (b, c) Representative Western blot and quantitative analysis of TXNIP. (d) qRT-PCR results of *TXNIP* mRNA. ^∗∗^*P* < 0.01*vs*. NC group, ^##^*P* < 0.01*vs*. LPS+control shRNA group (*n* = 6).

As shown in Figure 6(a), no fluorescence was observed in the NC and LPS groups. However, bright green fluorescence was observed in the hippocampus of each group of mice transfected with the AVV, indicating that the AAV successfully transfected the hippocampus and expressed the green fluorescent protein.

The qRT-PCR and Western blot results (Figures 6(b)–6(d)) showed that the hippocampal TXNIP mRNA and protein expression levels in the LPS and LPS+control shRNA groups were significantly upregulated compared to those in the NC group (*P* < 0.01). Compared to the LPS+control shRNA group, the TXNIP mRNA and protein levels in the LPS+TXNIP shRNA_1_ and LPS+TXNIP shRNA_2_ groups were significantly downregulated (*P* < 0.01).

### 3.6. TXNIP Knockdown Improved the 7-Day Survival Rate and Cognitive Dysfunction

**Figure 7:**
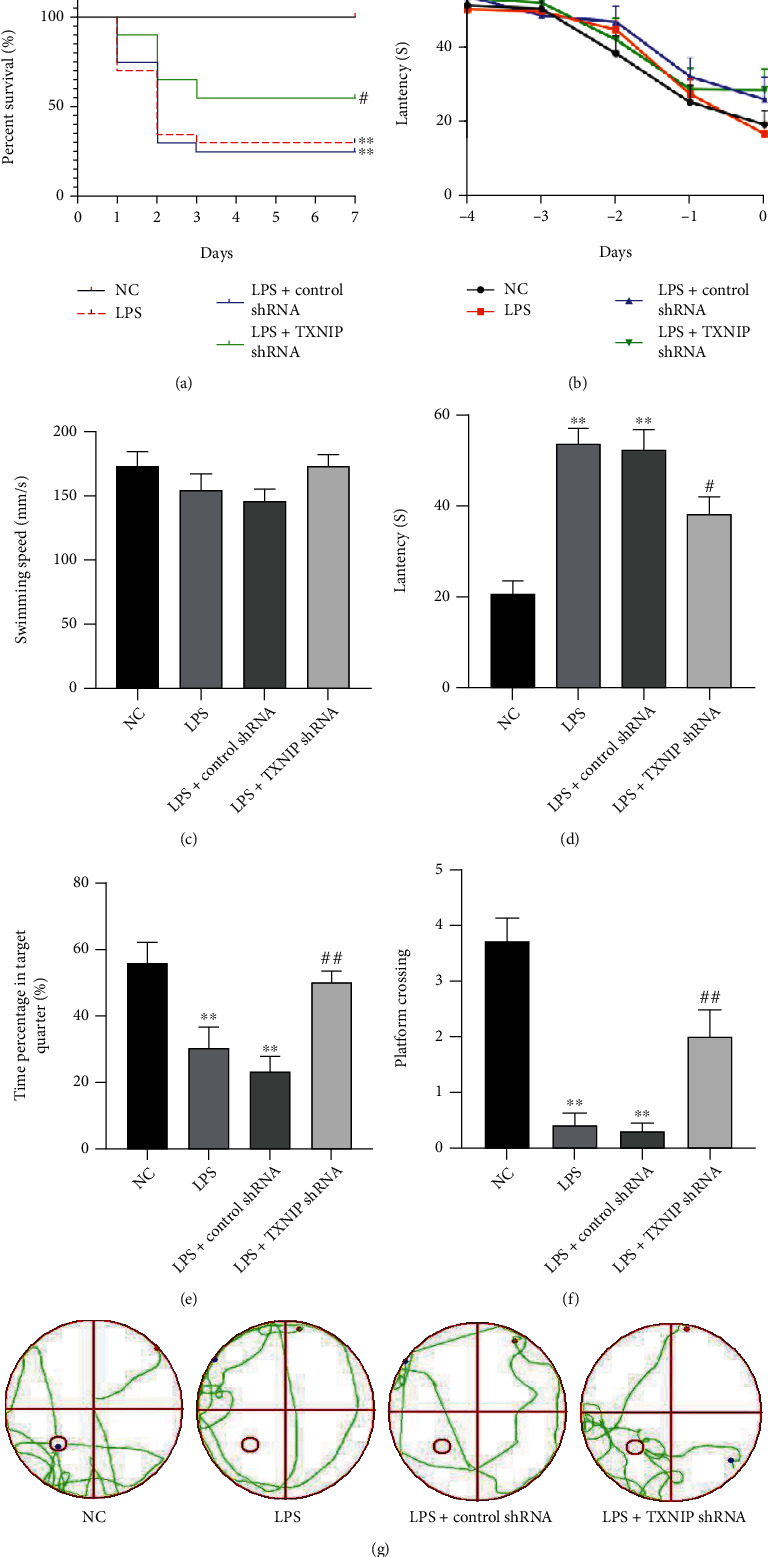
Effect of TXNIP knockdown on 7-day survival rate and cognitive function. (a) The Kaplan-Meier survival curve (*n* = 20). Cognitive function was assessed by the Morris water maze test. (b) The latency of each group before modeling (-4 d to 0 d). (c–g) The swimming speed, the latency of each group on the sixth day, percentage of stay time in the target quadrant, times of crossing the platform, and representative swim traces of each group in the space exploration experiment. The data are presented as mean ± SEM. ^∗∗^*P* < 0.01*vs.* NC group, ^##^*P* < 0.01, ^#^*P* < 0.05*vs.* LPS+control shRNA (*n* = 10).

As shown in Figure 7(a), the 7-day survival rate was 100% in the NC group. However, the 7-day survival rate in the LPS group and the LPS+control shRNA group was significantly lower than that in the NC group (*P* < 0.01). Conversely, the 7-day survival rate in the LPS+TXNIP shRNA group was significantly higher than that in the LPS+control shRNA group (*P* < 0.05).

Figures 7(b)–7(g) showed that the escape latency in each group was gradually shortened over the training time before modeling, and no statistically significant difference was detected among the groups (*P* > 0.05). However, the escape latency was prolonged, and the percentage of time in the target quadrant and the times crossing the platform were significantly reduced (*P* < 0.01) in the LPS and LPS+control shRNA groups compared to the NC group. Furthermore, compared to the LPS+control shRNA group, the mice in the LPS+TXNIP shRNA group showed a shorter escape latency and increased time in the target quadrant and times crossing the platform (*P* < 0.05 or *P* < 0.01). However, no significant difference was observed in the average swimming speed of the mice among groups (*P* > 0.05).

### 3.7. TXNIP Knockdown Attenuated Pathological Damage and Edema in the Brain Tissues

**Figure 8:**
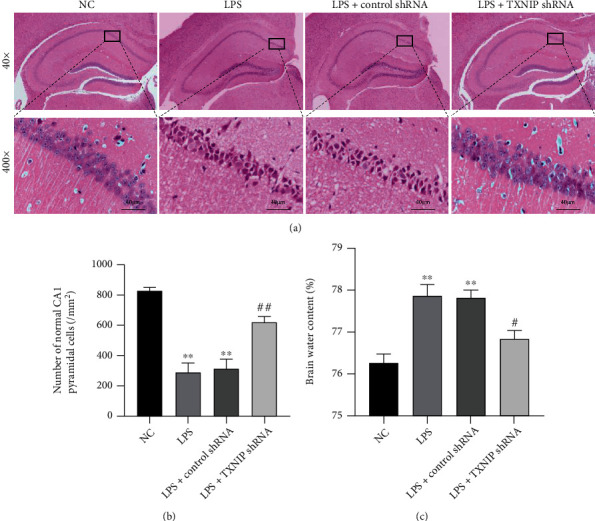
Pathological changes and encephaledema in the brain tissues. (a) HE staining in the hippocampal CA1 region (×400, scale bar = 40 *μ*m). (b) Statistics of the normal pyramidal cells in the hippocampal CA1 area. (c) The brain water content. The data are presented as the mean ± SEM. ^∗∗^*P* < 0.01*vs.* NC group; ^#^*P* < 0.05, ^##^*P* < 0.01*vs.* LPS+control shRNA group (*n* = 8).

The results of HE staining (Figure 8(a)) showed a regular morphology of the hippocampal CA1 region in the NC group. The morphologically normal pyramidal cells with clear nuclei and nucleoli were observed. However, many injured neurons, characterized by the shrunken appearance and dark staining, were observed after LPS injection.

Moreover, the mice in the LPS and LPS+TXNIP shRNA groups had significantly fewer normal neurons in the hippocampal CA1 area and higher brain water content in the whole brain tissues than those in the NC group at 24 h after LPS injection (*P* < 0.01). Compared to the animals in the LPS+control shRNA, normal neurons in the hippocampus were increased, and the brain edema was reduced significantly after TXNIP knockdown (*P* < 0.05 or *P* < 0.01) (Figures 8(b) and 8(c)), suggesting that TXNIP knockdown could reduce the brain damage in septic mice.

### 3.8. TXNIP Knockdown Reduced the Hippocampal Neuronal Apoptosis

**Figure 9:**
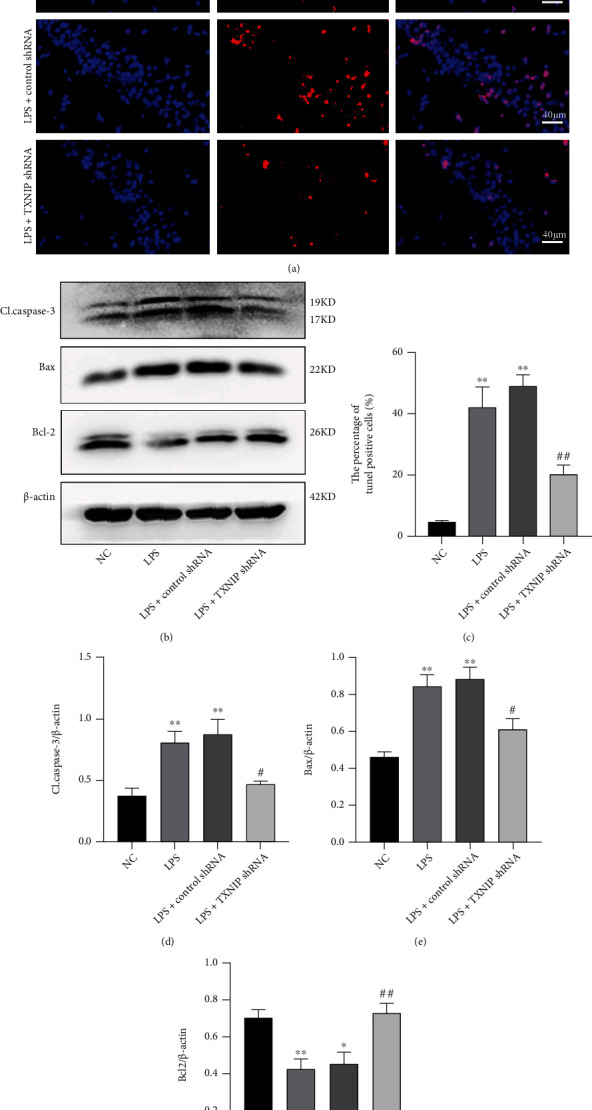
Neuronal apoptosis and expression of the apoptosis-related proteins in hippocampal CA1 of mice. (a) TUNEL (red) staining in the hippocampal CA1 area (×400) (scale bar = 40 *μ*m). (b) The bands of Bcl-2, Bax, and cleaved caspase-3. (c) Percentage of TUNEL-positive cells in the hippocampus. (d–f) Relative quantification of cleaved caspase-3, Bax, and Bcl-2. Data are presented as mean ± SEM. ^∗^*P* < 0.05, ^∗∗^*P* < 0.01*vs*. NC group; ^#^*P* < 0.05, ^##^*P* < 0.01*vs*. LPS+control shRNA group (*n* = 6-8).

The neuronal apoptosis in the hippocampal CA1 region of the brain was evaluated by the TUNEL method. No obvious apoptotic cells were found in the NC group, but those in the LPS and the LPS+control shRNA groups increased significantly (*P* < 0.01). These changes were alleviated by TXNIP knockdown and manifested as a decreased number of TUNEL-positive cells (*P* < 0.01) (Figures 9(a) and 9(c)).

The results of Western blot (Figure 9(b)) showed increased protein levels of the cleaved caspase-3 and Bax in the LPS and the LPS+control shRNA groups compared to the NC group (*P* < 0.01) but decreased levels in the LPS+TXNIP shRNA group compared to the LPS group (*P* < 0.05). On the other hand, the protein level of Bcl-2 was significantly lower in the LPS and LPS+control groups compared to the NC group (*P* < 0.01), but it increased in the LPS+TXNIP shRNA group compared to the LPS+control group (*P* < 0.01) (Figures 9(d)–9(f)).

### 3.9. TXNIP Knockdown Inhibited the Microglial Activation in the Hippocampus

**Figure 10:**
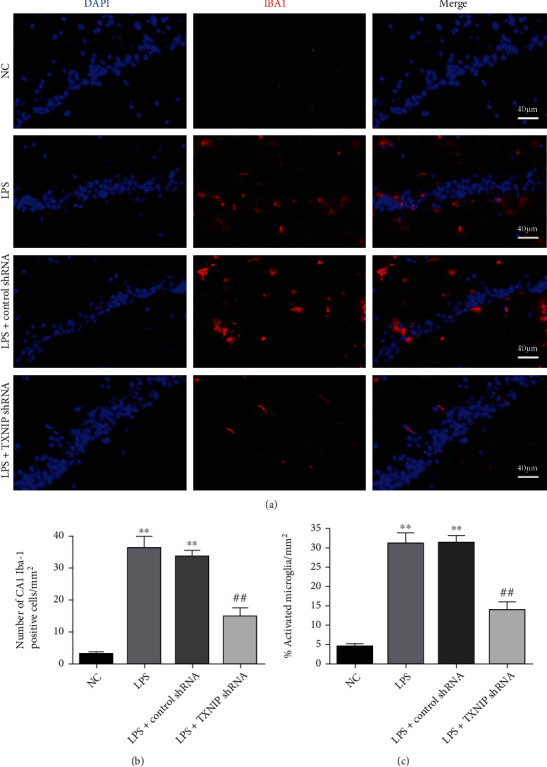
Effects of TXNIP knockdown on microglial activation. (a) Iba1 (red) immunofluorescence in the hippocampus (×400) (scale bar = 40 *μ*m). Nuclei were labeled with blue fluorescence (DAPI); Iba1 was labeled with red fluorescence. (b, c) Quantitative analysis of Iba1-positive cells and activated microglia in the hippocampus. ^∗∗^*P* < 0.01*vs*. NC group; ^##^*P* < 0.01*vs*. LPS+control shRNA group (*n* = 8).

To better understand the effect of TXNIP knockdown on the activation of microglia after sepsis, double immunofluorescence staining was performed with Iba1 and DAPI (Figure 10). Iba1 protein in the microglia was fluorescently labeled in the hippocampus of each group at 24 h after LPS injection. The microglia cells were small, and the synapses were slender and scattered in the cortex in the NC group. The number of Iba1-positive cells and the percentage of activated microglia were observed in the LPS and LPS+control shRNA groups accompanied by enlarged cell bodies and shortened synapses that accumulated in the hippocampus (*P* < 0.01). However, the number of Iba1-positive cells and the percentage of activated microglia in the hippocampus were significantly inhibited after TXNIP knockdown (*P* < 0.01).

### 3.10. TXNIP Knockdown Improved ROS, MDA, GSH-Px, and SOD Levels in the Hippocampus

**Figure 11:**
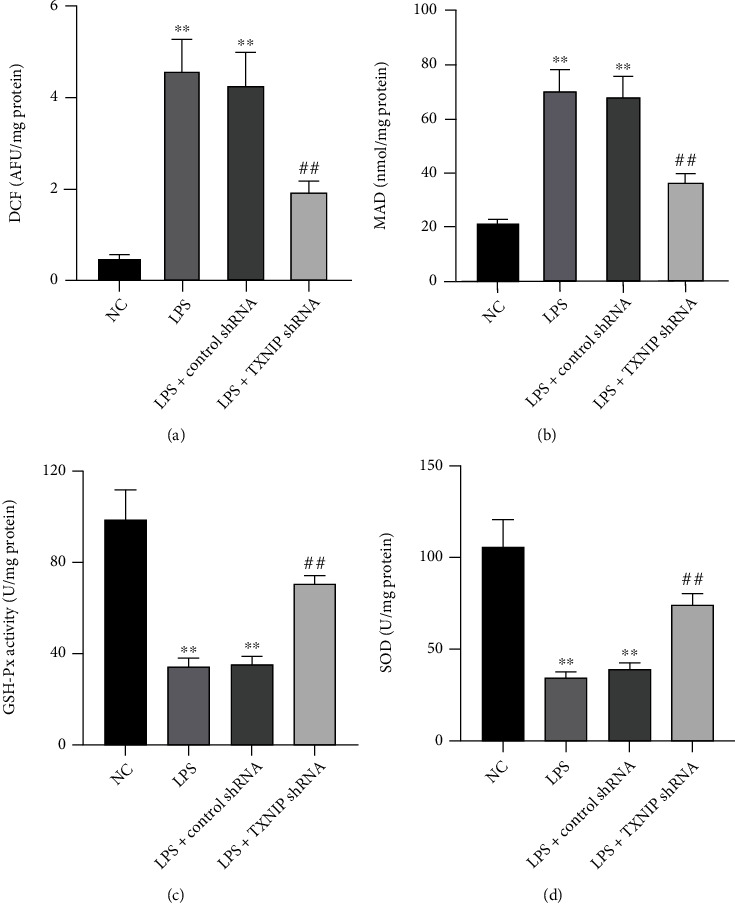
Effects of TXNIP knockdown on ROS, MDA, GSH-Px, and SOD levels. TXNIP knockdown significantly reduced the ROS and MDA levels and improved GSH-Px and SOD activities (a–d). The data are expressed as mean ± SEM. ^∗∗^*P* < 0.01*vs*. NC group; ^##^*P* < 0.01*vs*. LPS+control shRNA group (*n* = 8).

Hippocampal ROS, MDA, GSH-Px, and SOD levels are excellent indexes reflecting lipid peroxidation and the antioxidant ability of tissues. As shown in Figure 11, the levels of ROS (Figure 11(a)) and MDA (Figure 11(b)) were significantly higher (*P* < 0.01), while GSH-Px (Figure 11(c)) and SOD (Figure 11(d)) activities were markedly lower (*P* < 0.01) in the LPS group compared to the NC group. In addition, ROS and MDA levels were significantly lower (*P* < 0.01), and GSH-Px and SOD activities were markedly higher (*P* < 0.01) in the LPS+TXNIP shRNA group compared to the LPS+control shRNA group.

### 3.11. TXNIP Knockdown Reduced the Levels of Inflammatory Factors

**Figure 12:**
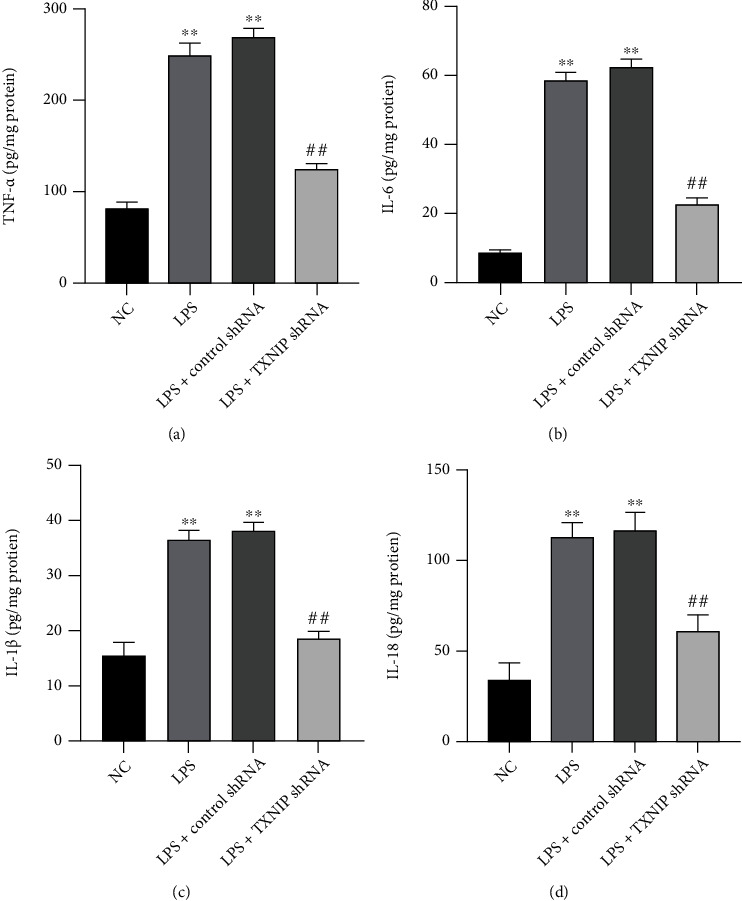
Changes in the inflammatory factors in the hippocampus. (a–d) The levels of TNF-*α*, IL-6, IL-1*β*, and IL-18 in the hippocampus, respectively. The values are presented as mean ± SEM. ^∗∗^*P* < 0.01*vs*. NC group; ^##^*P* < 0.01*vs*. LPS+control shRNA group (*n* = 10).

As presented in Figure 12, the levels of TNF-*α*, IL-6, IL-1*β*, and IL-18 were significantly increased in the LPS and LPS+control shRNA groups compared to the NC group (*P* < 0.01), while the levels of inflammatory factors were reduced in the LPS+TXNIP shRNA group compared to the LPS+control shRNA group (*P* < 0.01).

### 3.12. TXNIP Knockdown Inhibited the NLRP3/Caspase-1 Signaling Pathway

**Figure 13:**
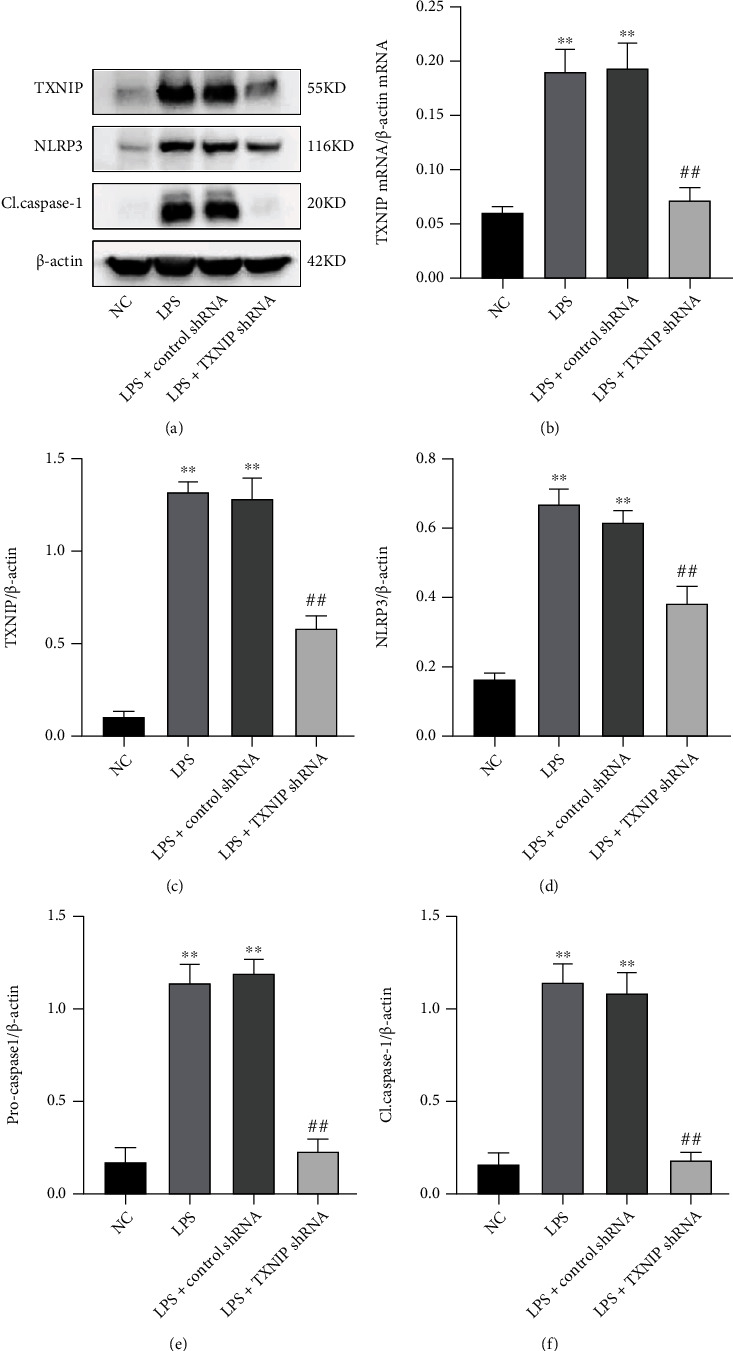
Expression of the TXNIP-NLRP3 signaling pathway in the hippocampus. (a) Bands of TXNIP, NLRP3, procaspase-1, and cleaved caspase-1 in the hippocampus. (b) qRT-PCR results of TXNIP mRNA. (c–f) Quantification of the TXNIP, NLRP3, procaspase-1, and cleaved caspase-1 levels in the hippocampus normalized to *β*-actin. The values are presented as mean ± SEM. ^∗∗^*P* < 0.01*vs*. NC group; ^##^*P* < 0.01*vs*. LPS+control shRNA group (*n* = 6).

As presented in Figure 13, compared to the NC group, the expressions of TXNIP, NLRP3, procaspase-1, and cleaved caspase-1 were significantly upregulated in the LPS and LPS+control shRNA groups (*P* < 0.01). Compared to the LPS+control shRNA group, the levels of these indicators were significantly downregulated in the LPS+TXNIP shRNA group (*P* < 0.01), indicating that TXNIP knockdown significantly inhibits the NLRP3/caspase-1 signaling pathway.

## 4. Discussion

The current study found the following. (1) The TXNIP and NLRP3 inflammasome expression levels were increased in the hippocampal CA1 region of the brain at 6, 12, and 24 h after LPS injection, especially at 24 h. (2) TXNIP knockdown significantly improved the 7-day survival rate, reduced the brain water content and brain injury, and improved the cognitive dysfunction of sepsis brain injury mice. (3) TXNIP knockdown inhibited the activation of microglia, downregulated the NLRP3/caspase-1 signaling pathway, and reduced the oxidative stress and the neuroinflammatory cytokine levels at 24 h post-LPS injection. (4) TXNIP knockdown significantly inhibited the hippocampal neuronal apoptosis and downregulated the expression of apoptosis-associated proteins cleaved caspase-3 and Bax, but upregulated the antiapoptotic protein Bcl-2. These results indicated that the TXNIP knockdown attenuated brain injury and cognitive decline in septic mice by suppressing oxidative stress and neuroinflammation.

Sepsis is a common complication of uncontrolled infection following severe trauma and other stress states, leading to shock, and is the main cause of ICU hospitalization and deaths worldwide [1, 2, 40, 41]. SAE is a diffuse brain dysfunction that occurs secondary to sepsis without overt central nervous system infection with the clinical manifestations of delirium, coma, seizures, and focal nervous system signs. A prospective autopsy study showed that ischemic lesions are found in the brains of all patients with SAE [10]. These ischemic lesions are mainly located in the brain areas that reduce the cerebral blood flow [2, 42]. The hippocampal CA1 region is extremely vulnerable to ischemia, hypoxia, and other harmful stimuli [43]. It plays a crucial role in learning and memory, and its destruction and dysfunction have been linked to cognitive deficits [30, 36, 44]. Therefore, combining those factors, the CA1 region was selected in the study rather than CA2, CA3, or dentate gyrus regions.

Lipopolysaccharide plays a significant role in Gram-negative bacterial infection and disease pathogenesis and has been widely used in the establishment of sepsis animal models [45]. The mice are remarkably less sensitive to the toxic or lethal effects of LPS. Thus, the dose of LPS, which leads to death in approximately half of mice (i.e., the LD50 dose) is about 1-25 mg/kg [46]. Interestingly, our preliminary experiments also showed that 7-day survival rates of mice gradually decreased with the different doses of LPS (5, 7.5, 15, or 20 mg/kg). LPS at 20 mg/kg caused total death in mice within 7 days, while 5 and 7.5 mg/kg did not cause appropriate septic brain damage (data not shown). Therefore, combining the above factors, we chose the dose of 15 mg/kg in the study based on our preliminary experiments and previous studies [47–49]. Previous studies have shown that the levels of proinflammatory cytokines, TNF-*α*, IL-1*β*, and IL-6, began to rise in 2-4 h after the intravenous or intraperitoneal injection of LPS, and these changes continued for several hours up to 24 h [45, 50]. In the present study, the mice manifested the sepsis symptoms such as upside-down hair, loose stools, hunched back, shortness of breath, and white viscous material around the eyes after the intraperitoneal injection of LPS at a dose of 15 mg/kg, and the 7-day survival rate was decreased significantly, while the expression levels of the TNF-*α*, IL-6, IL-1*β*, and IL-18 in the serum and hippocampus of mice after LPS injection were increased significantly. The cell arrangement in the hippocampus was disordered, the cytoplasm was deeply stained, and the nucleus was constricted. Moreover, the number of normal cone cells in the hippocampus decreased with prolonged injection time. These results indicated that septic brain damage could be induced in mice by an intraperitoneal injection of 15 mg/kg of LPS. It is reported that most damage of the brain might be caused around 6 to 48 h after sepsis, including gut origin sepsis [36, 51, 52]. The number of morphologically normal pyramidal cells with clear nuclei and nucleoli in the hippocampal CA1 region was counted according to many previous studies [36, 51]. However, damaged neurons with abnormal appearing condensed, pyknotic, and shrunken nuclei were not counted. Therefore, the results showed that LPS might reduce the number of morphologically normal pyramidal cells but not the total number of pyramidal cells (including cells with abnormal morphology) in the CA1 area within 24 hours and even 6 hours after sepsis. Since apoptotic cells cannot be identified well by hematoxylin-eosin staining, therefore, TUNEL staining was further performed. The results also showed that apoptotic cells in the CA1 area increased after LPS injection. This phenomenon is also consistent with many previous studies [30, 36, 53, 54].

The NLRP3 inflammasome is composed of NLRP3, ASC, and caspase-1 zymogen that are crucial components of the body's innate immune system. After activation, NLRP3 recruits ASC and caspase-1 zymogen and promotes the maturation and release of the inflammatory cytokines: IL-1*β* and IL-18 [19, 55]. Therefore, the inhibition of NLRP3 inflammasome activity has been suggested as a strategy for the treatment of neurological diseases [19]. The NLRP3 inflammasome promotes inflammation and apoptosis and is involved in the pathogenesis of SAE [51]. The hippocampus-dependent memory impairment induced by the cecal perforation (CLP) was accompanied by increased levels of NLRP3, caspase-1, and proinflammatory cytokines. The application of the NLRP3-specific inhibitor MCC950 improved cognitive deficits and reduced the NLRP3/caspase-1 pathway-induced neuronal pyrolysis in a mouse model of SAE [20]. A recent study also showed that the caspase-1 inhibition dramatically downregulated the pyroptosis, reduced the release of the inflammatory cytokines, protected the ultrastructure of the brain, and preserved the cognitive functions in the CLP-induced experimental sepsis [56]. In the current study, Western blot results showed that hippocampal NLRP3, procaspase-1, and cleaved caspase-1 were upregulated in the sepsis brain injury mice. Furthermore, we discovered that the expression of TXNIP in the hippocampus of septic mice also increased markedly. This increase occurred before the upregulation of NLRP3, procaspase-1, and cleaved caspase-1 expression levels. Therefore, we speculated that the TXNIP/NLRP3/caspase-1 pathway might mediate the LPS-induced septic brain damage.

In recent years, gene transfection technology has been widely used in various cell experiments and animal studies [34, 57]. In this study, the adeno-associated virus (AAV) vectors containing different interference fragments were injected into the lateral ventricle of the brain. Furthermore, virus was delivered at concentrations of 1.0 × 10^11^, 1.0 × 10^12^, and 1.0 × 10^13^ viral particles per mL in our preliminary experiments. After 4 weeks, the EGFP expressed by the AAV was observed in the hippocampus of the brain, indicating that AAV (1.0 × 10^13^ viral particles per mL) successfully infected the hippocampus. Importantly, the TXNIP mRNA and protein levels in the hippocampus were significantly reduced in the LPS+TXNIP shRNA_1_ and LPS+TXNIP shRNA_2_ groups, although TXNIP shRNA_1_ had the most obvious inhibitory effect. However, the control shRNA transfected with AAV as a vector did not affect the expression level of TXNIP. The behavioral pathological and inflammatory factors of the mice in the LPS+control shRNA group did not differ from those in the LPS group without virus injection, indicating that the AAV does not cause brain damage, and the regulatory effect of the shRNA transfected with the AAV on sepsis mice was mainly due to the transfection of various shRNAs. Unfortunately, it is difficult to make sure which one, the TXNIP gene or the whole compound, is exerting the role of neuroprotection in brain injury. We conjectured that reducing the gene of TXNIP might have potential protective effect, not the whole compound (TXNIP shRNA) in the present experiment based on previous studies [58, 59].

A strong link has been established between oxidative stress and inflammation [24]. TXNIP has been considered an endogenous negative regulator in the TRX system with a pivotal role in central system diseases, such as stroke, subarachnoid hemorrhage, and Alzheimer's disease [23, 25]. Ezetimibe reduces oxidative stress and neuroinflammation by activating the AMPK/Nrf2/TXNIP signaling pathway in rats with middle cerebral artery occlusion [60]. TXNIP shRNA was used to knock down TXNIP in experiment 3. Subsequently, TXNIP knockdown significantly improved the 7-day survival rate in sepsis mice, reduced brain edema and hippocampal pathological damage, and improved cognitive dysfunction. Sepsis is a systemic inflammatory response syndrome, often accompanied by the release of a large number of inflammatory cytokines, such as TNF-*α* and IL-6, in the central and peripheral nervous systems [7]. In this study, the release of proinflammatory cytokines, such as TNF-*α*, IL-6, IL-1*β*, and IL-18, was inhibited after TXNIP knockdown.

Oxidative stress also plays a key role in septic brain injury. The level of free radicals increases sharply during sepsis, accompanied by decreased activities of antioxidant enzymes. The MDA levels could reflect the severity of peroxidation in the body, while GSH-Px and SOD are crucial antioxidant factors [37, 38]. In the present study, ROS and MDA levels were increased evidently, while GSH-Px and SOD levels were decreased significantly as a consequence of decompensated activation of oxidative stress. TXNIP knockdown improved the alterations of oxidative stress. These findings demonstrated that TXNIP knockdown inhibits inflammatory responses, scavenges toxic free radicals, and regulates the activities of antioxidant enzymes, thereby exerting antioxidant effects. We also observed that the apoptotic rate of the hippocampal neurons in the TXNIP knockdown group was significantly lower than that of the sepsis mice without TXNIP knockdown. These results suggested that TXNIP is partially involved in the pathogenesis of sepsis brain injury.

The effect of the NLRP3 inflammasome on sepsis brain injury has been preliminarily studied in the CLP-induced mouse model, but the role of its regulatory protein TXNIP in the sepsis brain injury and the effects on the NLRP3 inflammasome remain unknown [20]. The microglia are the resident immune cells of the central nervous system and play a critical role in neuroinflammation [36]. In the central nervous system, NLRP3 is mainly expressed in microglia [39]. The maturation and release of IL-1*β* and IL-18 indicate NLRP3 inflammasome activation. The present study showed that TXNIP knockdown significantly inhibited hippocampal microglia activation in the sepsis mice, while the NLRP3 and cleaved caspase-1 protein expression levels and the inflammatory factors, IL-1*β* and IL-18, were significantly reduced in the hippocampus. The proapoptotic protein Bax and the antiapoptotic protein Bcl-2 mainly affect the release of the mitochondrial cytochrome C, thereby activating or inhibiting caspase-3 [61]. We also observed that TXNIP knockdown upregulated the level of Bcl-2 and downregulated the cleaved caspase-3 and Bax levels in the hippocampus of sepsis mice, thereby inhibiting neuronal apoptosis. Consistent with the previous study [62], caspase-1 catalyzed by the activated NLRP3 inflammasome promotes the release of IL-1*β* and IL-18 to regulate inflammation and apoptosis and the production of caspase-3.

Nevertheless, the present study has some limitations. Firstly, this study only evaluated the effect of TXNIP knockdown on brain damage, and hence, it is necessary to further evaluate the effect of TXNIP overexpression on brain damage in sepsis mice. Secondly, this study only explored the related mechanisms of the neuroprotective effect of TXNIP on sepsis mice with respect to oxidative stress and inflammatory response, while efficiency and other mechanisms need further study. Thirdly, the main goal/emphasis of the present study was to demonstrate the effects of TXNIP knockdown on sepsis-mediated brain injury and cognitive decline of animal through a new experiment and to investigate the possible mechanisms related to suppressing oxidative stress and neuroinflammation; we did not thoroughly investigate its dosage and some correlation. If this study might draw a conclusion that TXNIP had some diagnostic potential, its dosage should be studied. That is a tremendous work, and we will design other new experiments to address this question in the future. Finally, the clinical application of this study needs to be elucidated further.

## 5. Conclusion

In conclusion, the present study demonstrated that the TXNIP knockdown ameliorated sepsis-induced brain injury and cognitive decline by preventing oxidative stress and neuroinflammation in mice. Therefore, TXNIP might be a potential therapeutic target in SAE patients.

## Data Availability

The data used to support the findings of this study are included within the article and are available from the corresponding author upon request.
